# Thiolated Carboxymethyl-Hyaluronic-Acid-Based Biomaterials Enhance Wound Healing in Rats, Dogs, and Horses

**DOI:** 10.5402/2011/851593

**Published:** 2012-01-11

**Authors:** Guanghui Yang, Glenn D. Prestwich, Brenda K. Mann

**Affiliations:** ^1^Department of Medicinal Chemistry and Center for Therapeutic Biomaterials, University of Utah, 419 Wakara Way, Suite 205, Salt Lake City, UT 84108, USA; ^2^Department of Bioengineering, University of Utah, 72 S. Central Campus Drive, Rm. 2750, Salt Lake City, UT 84112, USA; ^3^SentrX Animal Care, Inc., 615 Arapeen Drive, Suite 110, Salt Lake City, UT 84108, USA

## Abstract

The progression of wound healing is a complicated but well-known process involving many factors, yet there are few products on the market that enhance and accelerate wound healing. This is particularly problematic in veterinary medicine where multiple species must be treated and large animals heal slower, oftentimes with complicating factors such as the development of exuberant granulation tissue. In this study a crosslinked-hyaluronic-acid (HA-) based biomaterial was used to treat wounds on multiple species: rats, dogs, and horses. The base molecule, thiolated carboxymethyl HA, was first found to increase keratinocyte proliferation in vitro. Crosslinked gels and films were then both found to enhance the rate of wound healing in rats and resulted in thicker epidermis than untreated controls. Crosslinked films were used to treat wounds on forelimbs of dogs and horses. Although wounds healed slower compared to rats, the films again enhanced wound healing compared to untreated controls, both in terms of wound closure and quality of tissue. This study indicates that these crosslinked HA-based biomaterials enhance wound healing across multiple species and therefore may prove particularly useful in veterinary medicine. Reduced wound closure times and better quality of healed tissue would decrease risk of infection and pain associated with open wounds.

## 1. Introduction

Cutaneous wound healing requires precise coordination of epithelialization, dermal repair and angiogenesis [1]. The healing microenvironment involves interactions among inflammatory cells, growth factors, cytokines, the extracellular matrix (ECM), and cells in the damaged tissue [2]. Epithelialization ultimately depends on the proliferative, migratory and differentiation abilities of keratinocytes. Hyaluronan (HA) has been shown to interact with signaling cascades that influence cell migration, proliferation, and gene expression [3, 4]. Further, HA is a ligand of CD44, whose receptor expression has also been found to correlate with reepithelialization [5]. The suggested link between the presence of increased HA in the wound bed and enhanced reepithelialization has led to the development of a range of HA-containing biomaterials as wound dressings [6, 7].

Chemical modification of HA offers the opportunity to create new biomaterials for reparative and regenerative medicine that are biocompatible and bioactive and possess physical properties optimized to a specific clinical use [8–10]. One end-user-oriented strategy involves deconstruction of the ECM to its simplest components, followed by reassembly for wound repair, tissue engineering, or growth factor delivery [11, 12]. In this approach, HA is first carboxymethylated followed by introduction of crosslinkable thiol residues [13] to produce thiolated carboxymethyl-HA (CMHA-S). This new HA derivative can be crosslinked with either a thiol-reactive crosslinker such as polyethylene glycol diacrylate, or by disulfide bond formation. The subsequently crosslinked hydrogel can also be dried to form a thin film [14]. Preliminary studies showed that crosslinked CMHA-S films could accelerate wound closure by  reepithelialization in acute full-thickness wounds in mice, and that the quality of the regenerated dermis was significantly better than in untreated wounds [12]. In addition, the CMHA-S films and gels have been shown to be effective in vivo for the treatment of injured vocal folds [15], for scar-free healing after endoscopic sinus surgery [16], for preventing stenosis after tracheal injury [17], and for preventing postsurgical adhesions [18, 19]. To date, however, no studies have examined the efficacy of these materials for veterinary wound care across multiple target species. This is particularly important given the fact that large animals tend to get severe wounds on their legs that can be difficult to treat, particularly on the lower limbs of horses where the wounds develop a large amount of exuberant granulation tissue, commonly called proud flesh. Such wounds are often debrided multiple times to remove the proud flesh; the resulting epithelial scar that forms is inferior to healthy epidermis, and the process is painful to the animal.

In these studies we first compared the ability of two formulations of CMHA-S, a viscous salvelike gel and a hydratable thin film, to accelerate and improve the quality of wound healing in a rat full-thickness bilateral excisional injury model. We then tested the ability of the hydratable thin films to accelerate wound healing in full-thickness injury models on the forelimbs of dogs and horses. These studies demonstrate the differences observed in rates of healing across multiple species, from small to large animals, and the effect of using a HA-based biomaterial during the healing process.

## 2. Materials and Methods

### 2.1. CMHA-S Synthesis

Thiolated carboxymethyl HA (CMHA-S) was prepared from HA (molecular weight (MW) 900 kilodaltons (kDa); Novozymes) by a modification to a method for preparing thiolated HA [13]. MW (340 kDa) was assessed using gel permeation chromatography and dynamic light scattering. CMHA-S with two levels of thiol modification—high thiol (7.5 × 10^−4 ^mmol thiol/mg CMHA-S) and low thiol (3.0 × 10^−4 ^mmol thiol/mg CMHA-S)—was synthesized. Thiol modification was assessed using 5,5′-dithio-bis(2-nitrobenzoic acid) (Ellman's reagent; Sigma-Aldrich).

### 2.2. PEGda Synthesis

Polyethylene glycol (PEG; MW 3350; Sigma-Aldrich) was acrylated as previously described [20], except that the resultant PEG-diacrylate (PEGda) was purified by dialysis.

### 2.3. CMHA-S Films and Gels

CMHA-S films were created by separately dissolving high-thiol CMHA-S in phosphate-buffered saline (PBS; pH 7.4) and PEGda in PBS. These solutions were then mixed and poured into a mold (2.4 × 3.7 cm) to a depth of 3 mm. The final concentrations of the CMHA-S and PEGda were 20 mg/mL and 8 mg/mL, respectively. The mixed solution formed a solid hydrogel within 15 min. Molds were kept at room temperature for 2 h, then placed in an oven for at least 12 h until the hydrogel dried to a thin film. Films were removed from the molds, placed in Tyvek pouches, and sterilized in a Sterrad 100S (Advanced Sterilization Products).

CMHA-S gels were created by dissolving low thiol CMHA-S in PBS to a final concentration of 10 mg/mL. The CMHA-S solution was filter-sterilized using a 0.8 *μ*m prefilter/0.2 *μ*m filter. The sterile solution was placed in a sterile stainless steel bowl, loosely covered, and mixed for 48 h under aseptic conditions to form a disulfide-crosslinked hydrogel.

### 2.4. Cell Proliferation Assay

Human neonatal epidermal keratinocytes (HEKn) were purchased from Cascade Biologics and cultured in complete serum-free keratinocyte medium (K-SFM) containing recombinant epidermal growth factor (rEGF) and bovine pituitary extract (BPE) (Invitrogen).

HEKn cells (4,000) were seeded in 100 *μ*l media in each well of 96-well flat-bottomed microplates and incubated at 37°C in 5% CO_2_ for 12 h. Thereafter, all the medium was changed with K-SFM containing CMHA-S at final concentrations of 0.01 mg/mL, 0.1 mg/mL, and 1 mg/mL. At 48 h, 20 *μ*l MTS (Promega) reagent was added to each well, and cells were further incubated for 2 h. The absorbance of the samples was measured using a 96-well plate reader (molecular devices).

### 2.5. Animals

The experimental protocol and animal care complied with the Guide for the Care and Use of Laboratory Animals [21]. Protocols for rats and dogs were approved by the Institutional Animal Care and Use Committee of the University of Utah; the protocol for horses was approved by an internal committee for the South Valley Large Animal Clinic.

#### 2.5.1. Rats

Eight-week-old female Sprague-Dawley rats (*n* = 10; Charles River Laboratories, Wilmington, MA) were used. All rats were quarantined at least seven days at the Comparative Medicine Center (CMC) of the University of Utah and were maintained on a daily 12 h light/12 h dark cycle.

After anesthesia induction (Isoflurane via inhalation, 2 L/min 2.5% total), the surgical field was shaved and prepared for aseptic surgery with iodine and alcohol swabs. Two full-thickness wounds (1.0 cm x 1.0 cm) were created on the dorsal side of each rat bilaterally. In the first of five rats, wounds were dressed with the CMHA-S gel on the left side and no treatment on the right side. Both sides were then covered with sterile gauze that was held in place by a bandage, and then secured with two 9 mm Autoclips. In the second group of five rats, CMHA-S film was applied to the wound on the left side with the right side wound serving as an untreated control, again held in place with sterile gauze, bandage, and Autoclips.

Animals were housed individually and fed a standard postoperative diet. Rats were photographed and then euthanized (CO_2_ at 100 kPa) at 7 days after injury. The skin of the wound area from four rats from each group was harvested and placed in 10% neutral buffered formalin for paraffin embedding in preparation for the subsequent histological analyses. Sections were taken and stained with hematoxylin and eosin (H&E).

#### 2.5.2. Dogs

Dogs (9 of mixed breed, gender, and age (2–5 years)) were purchased from a local animal shelter, examined by the staff veterinarian at the CMC at the University of Utah, and quarantined at least 21 days prior to surgery. On the afternoon prior to procedure, a section of hair was clipped and a Duragesic patch applied. After anaesthesia induction (Propofol, 6 mg/kg IV), the hair on both forelimbs in the surgical field was clipped.

The forelimbs of the dog were extended and an area measuring 2 cm in diameter was described on the dorsolateral aspect of each using a stencil and marking pen. The forelimbs were then prepped for aseptic procedure and a scalpel was used to create a full thickness skin defect using previously marked lines as a guide. Hemostatsis was achieved by direct pressure. Once hemostasis was achieved, a baseline photograph of the wound site was obtained. The wound on one forelimb received a CMHA-S film prior to dressing, while the contralateral forelimb received no film but received chlorhexidine ointment. The forelimb to receive the film treatment was randomly assigned. Following film or chlorhexidine, gauze was placed on all wounds, which were then wrapped with brown cling gauze, followed by sheet cotton, more cling gauze, and Vetrap (3 M). Elastikon was used at the top to secure the dressing. Following recovery from anesthesia, the animal was returned to kennel housing.

Bandages were checked daily. In the event a bandage was missing, the wound was redressed with film for treated wounds or just bandaging for control wounds. All bandages were changed every 7 days, at which time photographs of the wounds were obtained. The wounds were then redressed with either CMHA-S film or no film. After 21 days, bandages were removed, and the wounds were photographed. The staff veterinarian reviewed the wounds and determined if the dog was suitable for release to the adoption program. All dogs were deemed suitable and released to the adoption program.

#### 2.5.3. Horses

Horses (8 of mixed breed, gender, and age (3-5 years)) were purchased at auction, examined by a veterinarian at South Valley Large Animal Clinic and quarantined for 21 days prior to surgery. Horses were prepped the day prior to surgery in a manner similar to that described for dogs. On the day of surgery, horses were anesthetized (diazepam (0.02 mg/kg IV) and ketamine hydrochloride (2.2 mg/kg IV)) and full-thickness wounds were created on the dorsolateral apect of each metacarpus of the horses similar to the dogs, except that the wounds were 2 cm × 3 cm. As with the dogs, the wound on one forelimb of each horse received a CMHA-S film prior to dressing, while the contralateral forelimb received no film but received chlorhexidine. The forelimb to receive the film treatment was randomly assigned. Wounds were then dressed in the same manner as the dogs. Following recovery from anesthesia, the animal was returned to housing.

Bandages were changed every 8-9 days. Unlike the dogs, no bandages had been removed by the animals between timepoints. At bandage changes, the wounds were photographed, retreated with CMHA-S film or no film and rebandaged. At the end of the study, the animals were examined by a veterinarian, and all were deemed suitable for release to auction.

### 2.6. Measurement of Epithelial Thickness

Epidermal thickness was measured with the harvested rat tissue from stratum basale to stratum corneum at five equidistant sites. Three points were counted on each slide, and the mean thickness of the epidermis was determined [22].

### 2.7. Evaluation of Wound Size

Images of the wound area were obtained using a digital camera, and the dimensions of the wound area were measured using image analysis software (ImageJ, Scion Corp.). Wound area was then determined as a percentage of the initial wound area using the following formula: A_t_/A_0_ × 100 (%), where A_0_ is the wound area on day 0, and the A_t_ is the wound area on day t. For rats and dogs, A_0_ was determined from images taken immediately after wounding. For horses, A_0_ was taken to be the size of the template used to create the wound as pictures were not obtained immediately after surgery.

### 2.8. Statistical Analysis

Individual 95% confidence intervals of the means were calculated for each experiment. Means were determined to be significantly different at the 95% confidence level if the confidence intervals did not overlap. Reported *P* values were determined using Student's paired *t*-tests between treated and control wounds on each animal.

## 3. Results

### 3.1. Effect of CMHA-S on Proliferation of Epidermal Keratinocytes

**Figure 1:**
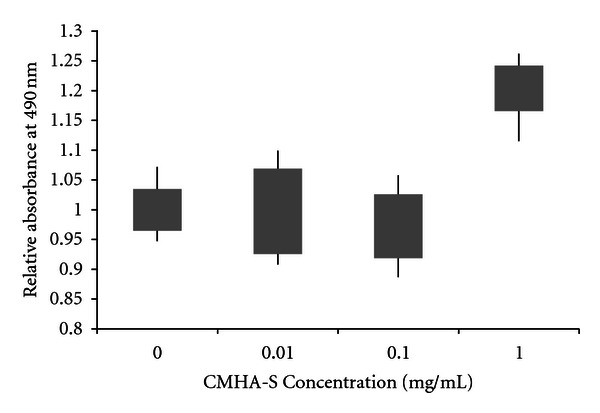
Effect of uncrosslinked CMHA-S on proliferation of epidermal keratinocytes. Absorbance was calculated relative to the mean absorbance for no CMHA-S in the medium. Bars represent 95% confidence intervals of the means; lines represent the range of values obtained.

To determine the effects of the uncrosslinked CMHA-S on the proliferation of epidermal keratinocytes, we incubated human neonatal epidermal keratinocytes (HEKn cells) with 0.01, 0.1, and 1.0 mg/mL CMHA-S in medium for 48 h.  Figure 1 shows that at low concentrations, CMHA-S has no net effect on keratinocyte proliferation. Interestingly, at 1.0 mg/mL, CMHA-S appears to significantly enhance keratinocyte proliferation relative to cells in control medium. 

### 3.2. Comparison of CMHA-S Films and Gels on Rat Wound Healing

On day 7 after wounding, gross appearance indicated the untreated wounds remained mostly unepithelialized. The wound area was filled with granulation tissue and was covered with a thick crust. In contrast, the sites treated with either CMHA-S film or gel showed suitable formation of healthy granulation tissue.

**Figure 2:**
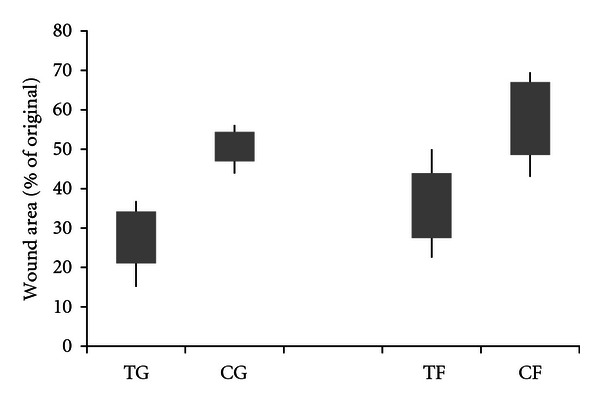
Comparison of CMHA-S film and gel on rat wound healing. Wound area with gel (TG) and film (TF) treatments compared with untreated controls (CG and CF, resp.) on day 7 after wounding. Bars represent 95% confidence intervals of the means; lines represent the range of values obtained.

The wound area for the CMHA-S gel-treated group was significantly smaller than that in untreated controls (*P* = 0.001). The wound area for the CMHA-S film treated group was also smaller than that in untreated controls on the same animal (*P* = 0.01). However, the wound areas of the film and gel-treated groups were not significantly different.  Figure 2 shows the fractional wound closure for these four groups.

**Figure 3:**
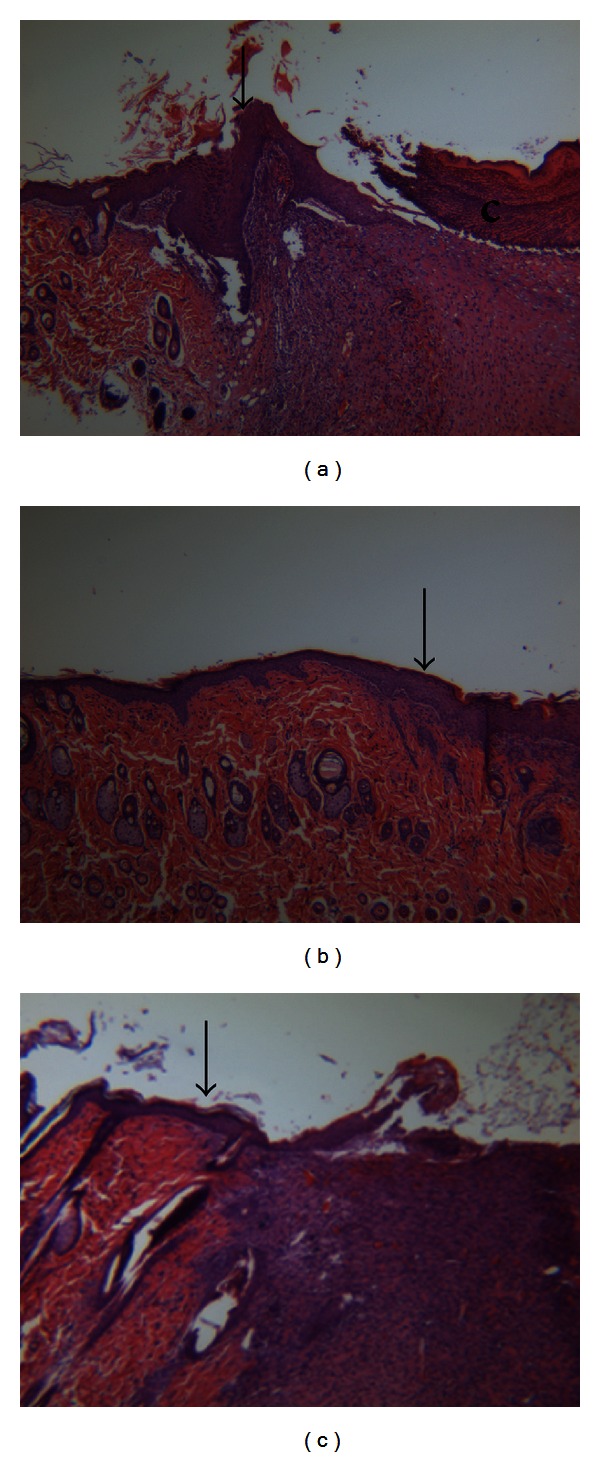
Histological staining of sections from rat wounds at day 7. (a) H&E staining of untreated wound; (b) H&E staining of wound treated with CMHA-S gel; (c) H&E staining of wound treated with CMHA-S film. Arrowheads indicate the interface between native rat skin and neos-skin; **c** = crust; H&E staining, 50x.

Histological analysis of the tissue also indicated that the untreated wounds were not fully epithelialized. Under the crust, a neo epidermis had begun to appear from the edge of the intact skin. As seen in Figure 3, H&E staining showed granulation tissue growth in each of the wounds. In contrast, thick and flat epidermis were observed in each of the wounds that had been treated with either the CMHA-S film or gel.

**Figure 4:**
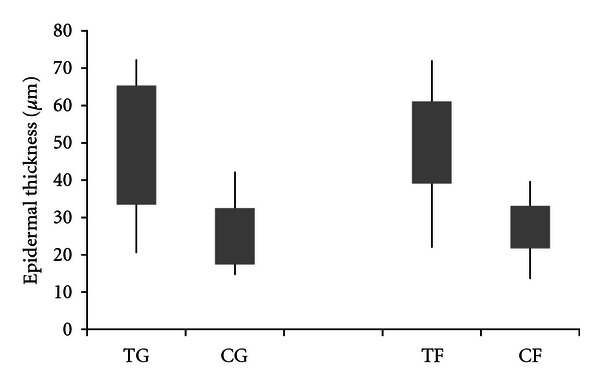
Epidermal thickness in CMHA-S gel-(TG) and film-(TF) treated wounds versus untreated controls (CG and CF, resp.). Bars represent 95% confidence intervals of the means; lines represent the range of values obtained.

Analysis of the histological data using ImageJ software (Figure 4) showed that the thickness of the epidermis in either the film or gel-treated groups was significantly greater than that in the untreated groups. However, the epidermal thickness of the film and gel treatment groups was not significantly different.

### 3.3. Effect of CMHA-S Films on Dog Wound Healing

**Figure 5:**
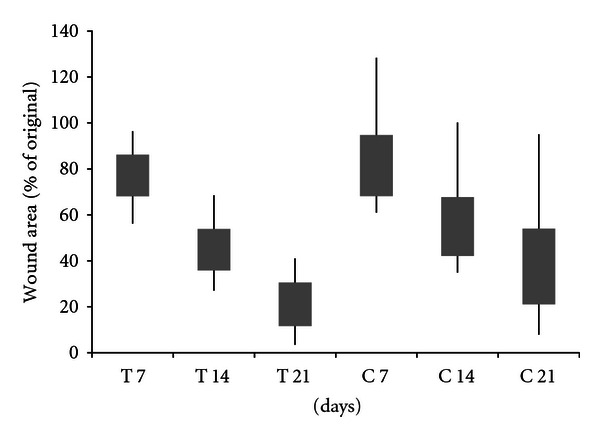
Area of CMHA-S film-treated (T) wounds on dogs compared to untreated controls (C) over the course of 21 days. Bars represent 95% confidence intervals of the means; lines represent the range of values obtained.

**Figure 6:**
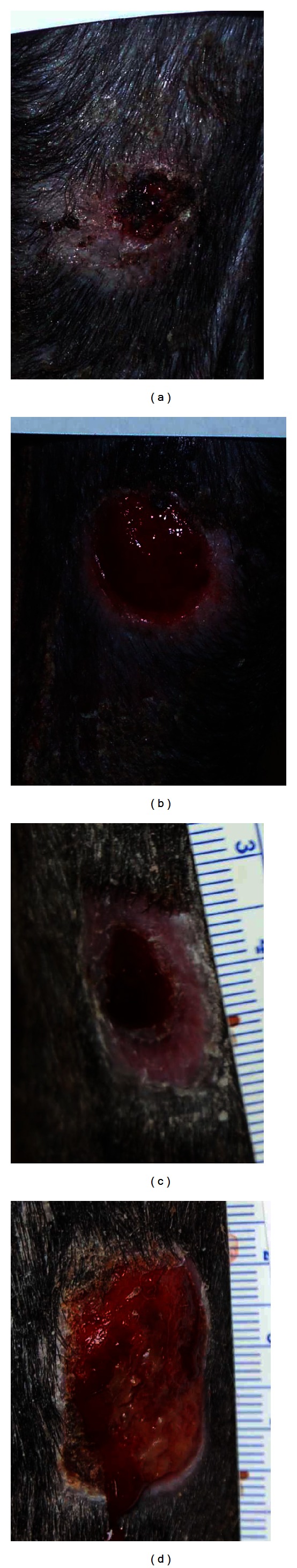
Photographs of CMHA-S film-treated wounds ((a), (c)) and untreated controls ((b), (d)) on the forelegs of a dog at 21 days ((a),(b)) and a horse at 26 days ((c), (d)). Note the amount of new epithelial tissue on the treated wounds, the raised granulation tissue with little epithelialization on the control wound of the dog (b), and the raised, yellowish exuberant granulation tissue on the control wound of the horse (d).

Wounds created on the forelimbs of dogs were photographed every 7 days, and the wound size determined using planimetry. Both treated and untreated wounds showed a steady progression of healing over the course of 21 days (Figure 5), with the area of the treated wounds trending toward being smaller than control wounds at both 14 and 21 days (*P* = 0.08 at 21 days). Additionally, the gross appearance of the wounds indicated that the treated wounds (Figure 6(a)) had more healthy granulation tissue and more epithelialization than control wounds (Figure 6(b)). It should be noted that some of the dogs were prone to removing their bandages between scheduled treatment dates. Therefore the number of times wounds were treated varied from dog to dog, since wounds were treated at each necessary rebandage. This led to some variability of wound closure when looking at individual animals. Variability was also expected due to the use of a mixed breed, gender, and age model used here.

### 3.4. Effect of CMHA-S Films on Horse Wound Healing

**Figure 7:**
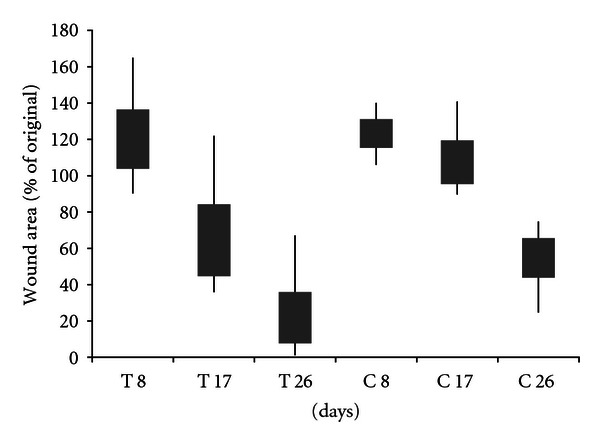
Area of CMHA-S film-treated (T) wounds on horses compared to untreated controls (C) over the course of 26 days. Bars represent 95% confidence intervals of the means; lines represent the range of values obtained.

Wounds created on the forelimbs of horses were photographed every 8-9 days, and the wound size were determined using imaging software. As with the dogs, the wounds of both treated and untreated wounds showed progressive healing over the course of 26 days (Figure 7). For the horses, wound areas are relative to the size of the template used rather than the postwounding area (as with the dogs and rats). There is retraction of the skin that occurs immediately following wounding such that the resultant wounds are actually larger than the size of the template. Thus, for the horses, the wound area on day 8 reflects some skin retraction of the wounds as well as some healing. The area of the treated wounds was significantly smaller than the control wounds at both 17 and 26 days (*P* = 0.0007 and *P* = 0.008, resp.). As with the rats and dogs, the gross appearance of the wounds indicated that the treated wounds (Figure 6(c)) had a healthier wound bed and more epithelialization than control wounds (Figure 6(d)). Unlike the dogs, the horses did not remove their bandages between the scheduled treatment times and thus all horses were treated for the same number of times. As with the dogs, there is expected variability due to the mixed breed, gender, and age model used with the horses as well.

## 4. Discussion

In this study, we examined the use of modified HA-based biomaterials to enhance wound healing. This is the first study that we are aware of to examine the use of a modified HA-based biomaterial to treat wounds on three animal species spanning a range of sizes, from rats to dogs to horses. Therefore, direct comparisons with other studies are limited. We first investigated the effect of our uncrosslinked modified HA, CMHA-S, on keratinocytes in vitro and found that it could promote keratinocyte proliferation. Since unmodified HA is also known to promote the proliferation and migration of keratinocytes [23], this indicated that the modifications made to HA here did not disrupt this activity.

We then explored the use of two formulations of crosslinked CMHA-S, a film and a gel, to promote the healing of full-thickness wounds on the backs of rats. The wound closure rate and histological examination showed that both treatments were statistically better than the controls, but did not reveal significant differences between the gel and film formulations. Both formulations have some advantages to them. For instance, the gel formulation is somewhat easier to apply to the wound bed, can be used for any size wound, and is more immediately accessible for aiding the wound healing process. However, the film rapidly hydrates once in contact with the wound by absorbing some blood and wound exudate that often contain beneficial growth factors and cytokines [24], which may be re-released as the film degrades. A different type of crosslinked HA sheet has previously been shown to promote epithelialization of rat wounds and was thought to be due in part both to the presence of HA and the absorption and release of cytokines from the wound surface [25]. Further, the film degrades more slowly due to a higher degree of crosslinking. In a study using esterified HA sheets in a human wound model, sheets with a slower degradation and thus a prolonged release of HA led to improved wound healing [26]. Both formulations used here, however, help maintain a moist wound healing environment. For the wound healing studies with dogs and horses, the film formulation was chosen for its advantages and the length of time between scheduled treatments.

The dogs and horses both had slower rates of wound healing than the rats. As the rate of wound healing will depend on not only the species but also the size of the wound and location (limb versus trunk) [24], a combination of these factors may have contributed to the different rates observed in these studies. Further, the larger animals had more variability in their wound closure than the rats, which may have been a function of using a different model (mixed breed, gender, and age) for the dogs and horses. However, we felt that such a model would better represent what one might expect in a clinical setting. Additionally, for the dogs there were some differences in the ultimate number of treatments some of the animals received as some animals were prone to removing their bandages. In these cases, we could have chosen to simply rebandage the wounds without treatment; however, this would have led to those dogs potentially receiving fewer treatments than others depending on when the bandage was removed, and still would have led to differences in the number of treatments among the dogs. In the end, the staff veterinarian indicated that if a bandage was removed by the animal in a clinical setting, the wound would be rebandaged with treatment and thus we chose to do so here.

The enhanced wound healing in the horses appeared to lead to less exuberant granulation tissue, a significant problem in this species, as indicated by gross examination of the wounds at the various time points. Iocono et al. have previously shown that repeated administration of a solution of hyaluronic acid to a wound bed leads to reduced granulation tissue and a matrix that more closely resembles that of fetal dermal connective tissue [27]. Thus, the extended release of CMHA-S through degradation of the crosslinked film here may lead to a reduction in granulation tissue.

Regardless of the species, the presence of the crosslinked CMHA-S material enhanced the rate of wound healing and appeared to enhance the quality of the tissue. A similar CMHA-S gel has been used to treat corneal wounds in rabbits, resulting in faster healing and a thicker epithelial layer with the treated wounds compared to control wounds [28]. Although the studies presented here were done on acute, surgically created wounds, they indicate the potential for using CMHA-S biomaterials in veterinary medicine, and that they may be used across multiple species to enhance and accelerate the wound healing process. A faster wound healing rate would also lead to decreased risk of infection due to having an open wound and decreased pain for the patient. Further studies have recently been done on horses to examine the use of single versus multiple applications of material as well as gel versus film in this species (L.A. Dahlgren et al., unpublished data).
